# EVALUATE THE IMPACT OF INTERDISCIPLINARY EDUCATION AND TELEHEALTH PRIMARY CARE: QUALITY AND EFFICIENCY

**DOI:** 10.1093/geroni/igad104.2063

**Published:** 2023-12-21

**Authors:** Ji Yoo, Jay Shen

**Affiliations:** Kirk Kerkorian School of Medicine at UNLV, Las Vegas, Nevada, United States; School of Public Health, UNLV, Las Vegas, Nevada, United States

## Abstract

In a provider shortage area, an Interprofessional Education and Telehealth Primary Care (IPETC) for geriatrics workforce was developed in 2020. To analyze the impact of IPETC on quality and efficiency of care, we measured quality outcomes defined by the CMS defined quality measures - dementia caregiver education/referral and advance care plan, and efficiency outcomes by estimating healthcare cost savings from reducing hospitalization. Two-hundred forty-four community-dwelling adults aged 60 to 97 with mild to moderate dementia were selected in an urban safety-net primary care clinic. Propensity - demographics and comorbidity was matched. Main outcomes were (A) number of hospitalization and 90-day rehospitalization rate, hospitalization-related healthcare cost estimates from the State Inpatient Dataset using the ICD-10 codes of principal diagnosis and hospital length of stay between January-December 2021. One-hundred twenty-two patients were cared by primary care providers who received the IPETC;122 patients were by those who did not have the IPETC; (2) CMS quality measures were compared between 2019 (baseline) and 2021 of patients cared by providers with IPETC. Number of hospitalization with IPETC was fewer than those without IPETC (0.72 vs. 1.38, p < .01). Number of 90-day rehospitalization with IPETC was fewer than those without IPETC (0.14 vs. 0.30, p < .01). An average cost-saving of $20,289 per patient was observed among those with IPETC than those without IPETC (p < .001). CMS quality measures improved from 15.6% to 51.0% in dementia caregiver education/referral; 10.2% to 24.7% in documented advance care plans.

